# French guidelines for the management of malignant hyperthermia

**DOI:** 10.1186/1471-2253-14-S1-A26

**Published:** 2014-08-18

**Authors:** René Krivosic-Horber

**Affiliations:** 1 Malignant Hyperthermia Unit, Academic Unit Anaesthesia, University Hospital Lille, Lille, 59037, France

## Background

Malignant hyperthermia (MH) is a complication of anaesthesia appearing as an acute potentially lethal hypermetabolic state in people carrying a genetic anomaly expressed in the skeletal muscle. Clinical guidelines aim to help health professionals and patients make the best decisions about treatment or care for a particular condition or situation. The guidelines are typically written in statement form by a reputable organization. The authors of guidelines review the research literature and take advice from experts to gather the current evidence on which to base the recommendations in a guideline. Doctors, nurses and other health care professionals are encouraged to follow clinical guidelines where appropriate [http://www.patient.co.uk/guidelines.asp].

The French Society of Anesthesiology and Intensive Care (SFAR) decided in 2012 to ask the French MH Group (FMHG) to write Guidelines for the management of Malignant Hyperthermia. The FMHG gathers the professionals in charge of the Five MH Centers involved in the diagnosis of MH by performing in vitro contracture test (IVCT), genetic analysis and/or an expert advice. Pr Renée Krivosic-Horber, Dr. Anne-Frédérique Dalmas Centre HM Lille, Pr Yves Nivoche Centre HM Robert Debré Paris, Pr Jean-François Payen Chu Grenoble, Pr Joël Lunardi, Madame Nicole Monnier, Pr Julien Faure Centre de Biologie Moléculaire CHU de Grenoble, Dr Alexandre Moerman Génétique Clinique CHRU de Lille, Dr Thierry Girard MH Center Basel Suisse was asked to give his opinion as a french speaking international expert.

Three meetings and many exchanges by mail between the experts and with the colleagues in charge of the Guidelines Board of the SFAR were necessary to obtain a consensus from all the participants. Eventually the text was published on the site of the SFAR in October 2013. http://www.sfar.org/article/1080/recommandations-d-rsquo-experts-pour-le-risque-d-rsquo-hyperthermie-maligne-en-anesthesie-reanimation-sfar-crc-12-septembre-2013.

Dr. Krivosic-Horber has been invited to present the Guidelines with a written paper in the annals, at the annual meeting of the SFAR in Paris in September 2014.

Index of the Guidelines:

1) Screening of the risk of MH susceptibility during the “consultation d’anesthesie” (which is legally mandatory in France).

2) How to do the diagnosis of MH susceptibility (IVCT or Genetic?)

1) How to perform an anesthesia free of MH risk.

2) Management of the MH crisis: presented as a poster with diagnosis and treatment on one side and stock and preparation of dantrolene on the other side.

3) What to do after the crisis.

4) The addresses of the MH centers and a map showing the type of diagnosis they can provide.

